# Supplementary material for: Effects of Positive Reinforcement Training and Novel Object Exposure on Salivary Cortisol Levels under Consideration of Individual Variation in Captive African Elephants (Loxodonta africana)
Source: Animals (Basel). 2021 Dec 10;11(12):3525. doi: 10.3390/ani11123525 (PMC8698154; doi:10.3390/ani11123525)
Supplement: Supplementary file 1 [file animals-11-03525-s001.zip › animals-1429343-supplementary.pdf]

Supplemental materials

Table S1. Number of saliva samples collected in each situation (positive-reinforcement-training [PRT], novel object exposure in the first study period [NOV2016] and in the second study period [NOV2017]), animal (sorted by zoo, then sex and age class) and sampling time as well as total number of samples per animal and situation.

Situation/ study period	Animal-ID	Number of samples per sampling time [min]									
		0	10	15	20	30	40	45	50	60	Total
PRT	AR	3	3	-	3	3	3	-	3	3	21
	ZI	3	3	-	3	3	3	-	3	3	21
	TA	3	3	-	3	3	3	-	3	3	21
	SA	3	-	3	-	3	-	3	-	3	15
	SW	3	-	3	-	3	-	3	-	3	15
	TI	3	-	2	-	3	-	3	-	3	14
	TU	3	-	3	-	3	-	3	-	3	15
	SF	3	-	3	-	3	-	3	-	3	15
	CH	3	-	3	-	3	-	3	-	3	15
	KB	3	-	3	-	3	-	3	-	3	15
NOV2016	AR	3	3	-	3	3	3	-	3	3	21
	ZI	3	3	-	3	3	3	-	3	3	21
	TA	3	3	-	3	3	3	-	3	3	21
	SA	3	-	2	-	2	-	3	-	3	13
	SW	3	-	3	-	3	-	3	-	2	14
	TI	3	-	3	-	2	-	3	-	3	14
	TU	3	-	2	-	3	-	3	-	3	14
	SF	3	-	3	-	3	-	3	-	3	15
	CH	3	-	3	-	3	-	3	-	3	15
	KB	3	-	3	-	3	-	3	-	3	15
NOV2017	AR	6	6	-	6	6	3	-	3	3	33
	ZI	6	6	-	6	6	3	-	3	3	33
	TA	6	6	-	6	6	3	-	3	3	33
	SA	5	-	6	-	6	-	3	-	3	23
	SW	5	-	5	-	6	-	3	-	3	22
	TI	5	-	4	-	6	-	3	-	3	21
	TU	6	-	5	-	5	-	3	-	3	22
	SF	6	-	6	-	6	-	3	-	3	24
	CH	6	-	6	-	6	-	3	-	3	24
	KB	6	-	6	-	6	-	3	-	3	24

Table S2. Fixed effects resulting from the linear mixed model examining the effect of experimental (sampling time [linear term]: 0, 10, 15, 20, 30, 40, 45, 50, 60 min, sampling time sq [quadratic term: $(\text{sampling time} - 30)^2$], situation: PRT = positive-reinforcement-training, NOV = novel object exposure, study period: 2016, 2017) and individual (age class, sex, zoo, see Table 1) factors on the salivary cortisol levels of ten captive African elephants (*n* = 589). Salivary cortisol concentrations were $\log_{10}$-transformed to satisfy the model assumptions (see 2.5). Animal-ID (*n* = 10) and trial nested in animal-ID (*n* = 60) were included as random effects.

**All animals/ PRT and NOV**					
**Fixed effect**	**Estimate**	**SE**	**df**	**t**	**p**
**Intercept**	**-0.51**	**0.15**	**6.58**	**-3.47**	**.012**
Sampling time	-3.59E-05	3.80E-04	540.70	-0.09	.925
Sampling time sq	-7.45E-06	2.06E-05	524.90	-0.36	.718
**Situation**	**-0.14**	**0.02**	**519.60**	**-6.74**	**< .001**
**Study period**	**-0.11**	**0.02**	**568.60**	**-5.89**	**< .001**
Age class	*0.10*	*0.04*	6.02	*2.37*	*.055*
**Sex**	**0.19**	**0.07**	6.07	**2.56**	**.042**
Zoo	0.06	0.04	5.86	1.55	.173

Note: Two-sided t-test (Satterthwaite's method) were used to test significance of fixed effects. Statistical significance ($p < .05$) in bold, results approaching significance ($p < .1$) in italics.

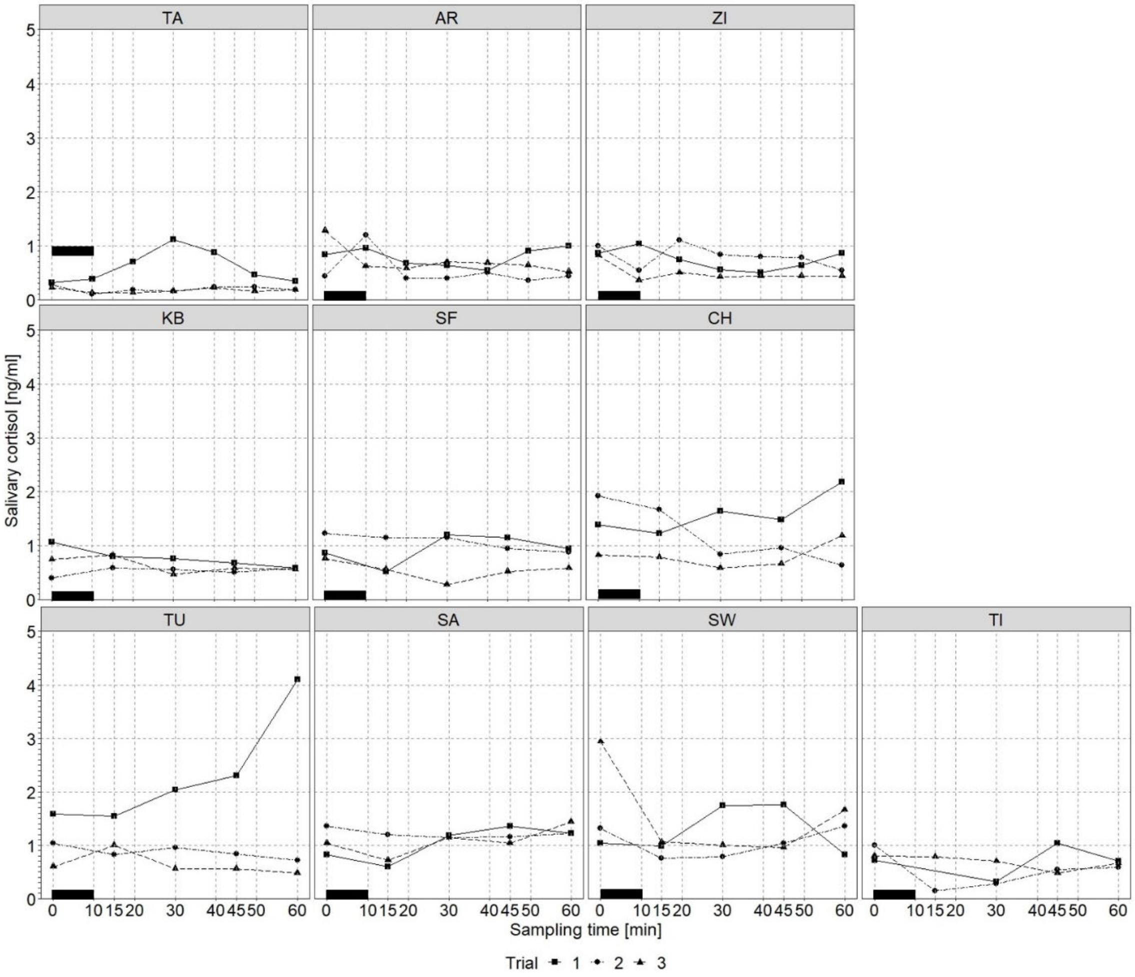

Figure S1. Salivary cortisol profiles of the three trials of positive-reinforcement-training (PRT) in the ten animals studied. Black bars indicate the time period in which PRT occurred. Animals in each row were housed in the same zoo. Animals in the first column were male, those in the remaining columns female elephants. Information on animals is given in Table 1 (main manuscript).

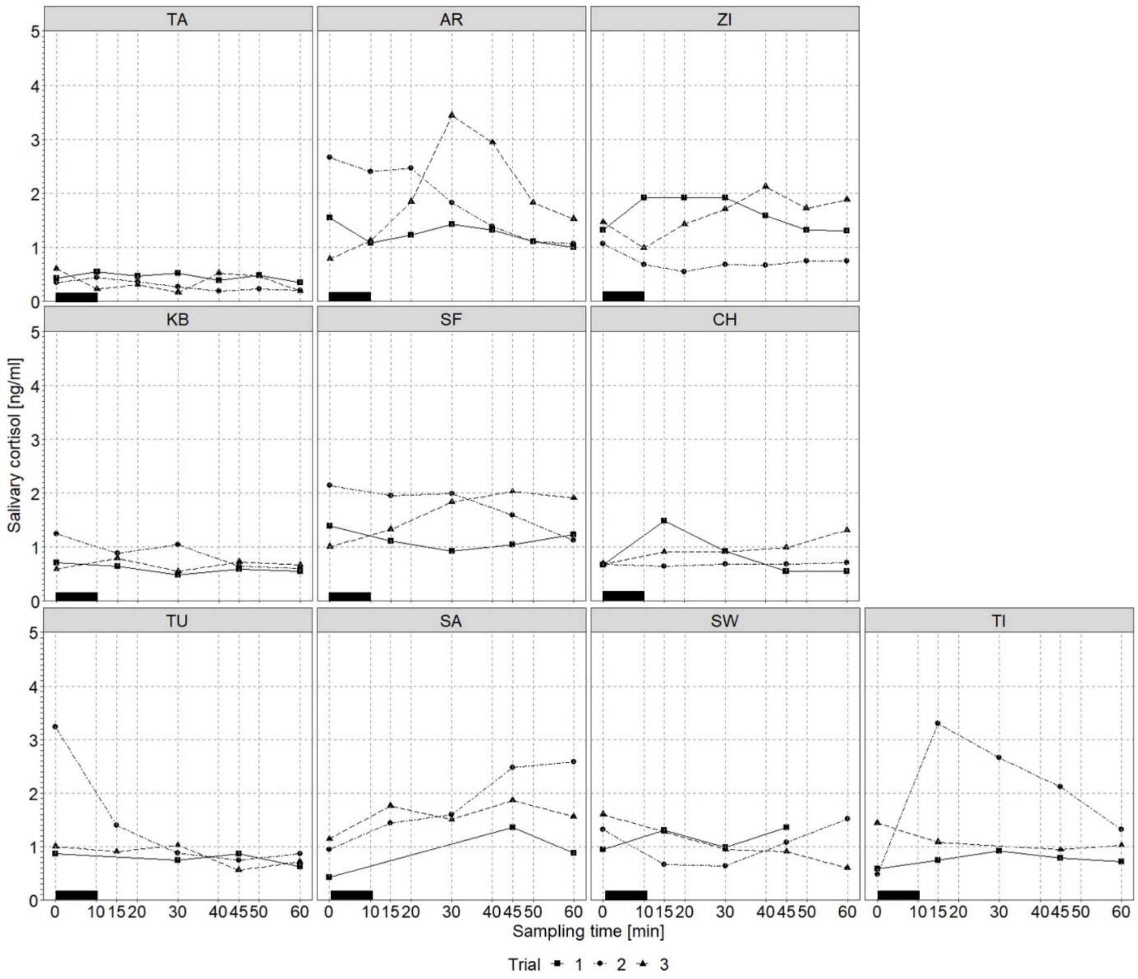

Figure S2. Salivary cortisol profiles of the three trials of novel-object exposure (NOV) in the first study period (2016) in the ten animals studied. Black bars indicate the time period in which NOV occurred. Animals in each row were housed in the same zoo. Animals in the first column were male, those in the remaining columns female elephants. Information on animals is given in Table 1 (main manuscript).

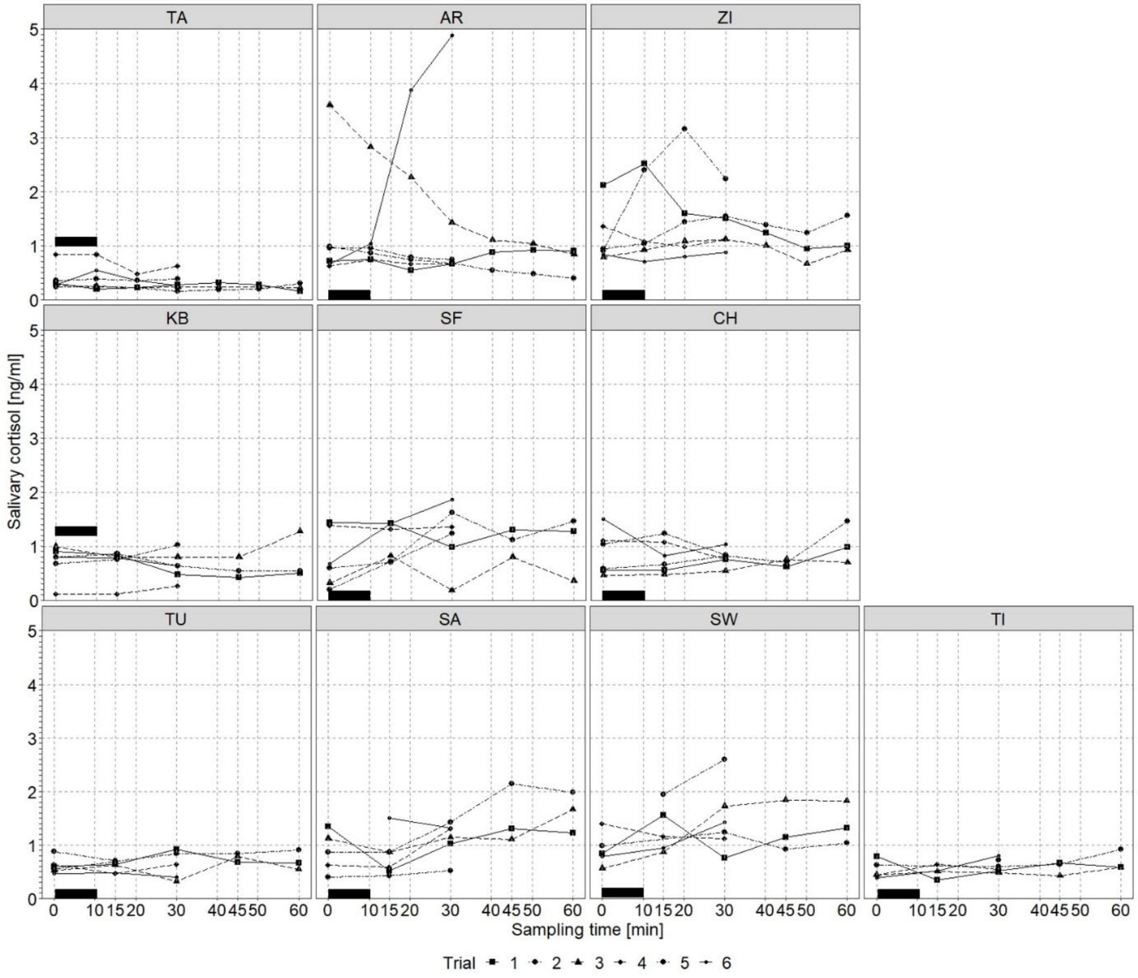

Figure S3. Salivary cortisol profiles of the six trials of novel-object exposure (NOV) in the second study period (2017) in the ten animals studied. Black bars indicate the time period in which NOV occurred. Animals in each row were housed in the same zoo. Animals in the first column were male, those in the remaining columns female elephants. Information on animals is given in Table 1 (main manuscript).
